# “Salus Populi” Not “Suprema Lex” (in Texas)

**Published:** 1899-04

**Authors:** 


"SALUS POPULI" NOT "SUPREMA LEX" (In Texas).—The Twenty-sixth Legislature will adjourn some time this month. The bill to create a State Board of Health, or some other institution for the better protection of the public health, has been quietly pigeon-holed and will not be acted on. This bill was prepared by a committee appointed by the Texas State Medical Association, and presented to the Legislature as an expression of the views of that body, —and, as it was said, in accordance with the wishes of the Governor, who expressed a desire to know the sentiment of the medical profession on the subject of sanitary legislation. It provides for a system of collection and preservation of the vital statistics of the State, without which no progress can ever be made in sanitary reform; and for the investigation and removal of the causes of those indigenous disease, such as meningitis—now prevalent—typhoid fever, pneumonia, etc., which annually destroy thousands of lives—more than are lost by all the epidemics of yellow fever that ever occur (and only six have occurred in Texas in the last half century). Meantime, the Legislature, duly impressed with the value of cattle and cotton, lost no time in making special appropriations for the investigation of the cattle disease called "black leg," and for investigating and "stamping out" (I hate that word) the cotton-boll worm. When it is recalled that cotton is worth five cents a pound, it will be seen what value is apparently placed on human life; it is not worth saving.

As to vital statistics, a child may be born in Texas, grow to manhood, and die, and there will be no evidence that could be used in law to show that he was ever born, ever lived or ever died. During life, should he be heir to an estate, he could never establish the fact that he was ever born; or if so, that his father and mother were ever married.

---

Death of Mr. Parke, senior member and organizer of the great house of Parke, Davis & Co.—Mr. Henry C. Parke, the head of this well known firm, died recently, and the announcement will be received with profound regret throughout the commercial world. His great enterprise and his great success in seconding every legitimate advance in therapeutics on the part of the medical profession are well known to physicians all over the world, and the firm was long ago recognized as a friend and able ally of the regular profession. His many private charities are known only to a few, but all unite in pronouncing him a benefactor of his race, and we feel assured that the world has profited by his having lived.

---

Lambert and Listerine Forever.—The Lambert Pharmacal Co. have sent us a nice little *souvenir* that is either a paper cutter or a stiletto, just as occasion may require, with "Listerine" on the handle, "lest we forget."

